# An Additional Electrodiagnostic Tool for Ulnar Neuropathy: Mixed across the Elbow

**DOI:** 10.1055/s-0040-1714742

**Published:** 2020-08-26

**Authors:** Drew B. Parkhurst, Michael T. Andary, John W. Powell

**Affiliations:** 1 Department of Physical Medicine and Rehabilitation, Michigan State University, Lansing, Michigan, United States

**Keywords:** electrodiagnostic testing, ulnar neuropathy, elbow, nerve conduction velocity

## Abstract

**Background** Diagnosing ulnar neuropathy at the elbow (UNE) remains challenging despite guidelines from national organizations. Motor testing of hand intrinsic muscles remains a common diagnostic method fraught with challenges.

**Objective** The aim of the study is to demonstrate utility of an uncommon nerve conduction study (NCS), mixed across the elbow, when diagnosing UNE.

**Methods** Retrospective analysis of 135 patients, referred to an outpatient University-based electrodiagnostic laboratory with suspected UNE between January 2013 and June 2019 who had motor to abductor digiti minimi (ADM), motor to first dorsal interosseus (FDI), and mixed across the elbow NCS completed. To perform the mixed across the elbow NCS, the active bar electrode was placed 10-cm proximal to the medial epicondyle between the biceps and triceps muscle bellies. The median nerve was stimulated at the wrist followed by stimulation of the ulnar nerve at the ulnar styloid. The difference between peak latencies, labeled the ulnar-median mixed latency difference (U-MLD), was used to evaluate for correlation between the nerve conduction velocities (NCV) of ADM and FDI.

**Results** Pearson *r* -values = −0.479 and −0.543 ( *p* < 0.00001) when comparing U-MLD to ADM and FDI NCV across the elbow, respectively. The negative *r* -value describes the inverse relationship between ulnar velocity across the elbow and increasing U-MLD.

**Conclusion** Mixed across the elbow has moderate–strong correlation with ADM and FDI NCV across the elbow. All three tests measure ulnar nerve function slightly differently. Without further prospective data, the most accurate test remains unclear. The authors propose some combination of the three tests may be most beneficial when diagnosing UNE.

Ulnar neuropathy at the elbow (UNE) is a common site for compression in the arm, second only to median mononeuropathy at the wrist. 1 2 Relying on sensory symptoms to secure a diagnosis is inherently problematic as nearly 40% of subjects with paresthesias in the ulnar distribution have been subsequently diagnosed with carpal tunnel syndrome. 3 Current techniques to evaluate UNE are not as sensitive as diagnosing other mononeuropathies in the upper extremity, with a high risk of false negatives especially with mild nerve injury. 3 4

The poor sensitivity can, at least partially, be explained by numerous pitfalls that have been described when assessing motor nerve conduction studies (NCS) of the ulnar nerve. Such aspects that limit utility include, measurement error, selective fascicular involvement, challenging data interpretation, and inability to adequately localize the lesion. 1 4 5 6 Measurement variability (standard deviation) of the across elbow distance increases when the elbow is flexed. 7 The absence of weakness or atrophy in muscles innervated by the ulnar nerve is another important factor that potentially limits the functionality of electrodiagnostic testing as these patients have more subtle abnormalities. 1 4 8 9 Interestingly, previous research indicates that subclinical axon loss can still occur despite patient's description of purely sensory symptoms. 4 Due to these numerous problems, a multiorganizational summary statement regarding identifying ulnar neuropathy recommends using multiple electrodiagnostic studies that agree to diagnose UNE rather than one isolated result. 10

A unique NCS measuring a mixed compound nerve action potential (CNAP) across the elbow has been described using two different techniques. 2 11 One technique still required elbow flexion, which did not solve issues with measurement error and only included 10 symptomatic patients. 11 The goal of this present study was to establish a correlation between this uncommon NCS, mixed across the elbow, and other more common ulnar motor NCS to further aid diagnosing UNE. Specifically, the slower ulnar velocity across the elbow should correlate with a larger difference between the distal latencies of the ulnar and median nerves.

## Methods

### Data Collection

Prior to data collection, this study was approved by the Institutional Review Board at both Michigan State University and McLaren Healthcare with a waiver for informed consent and in accordance with The Code of Ethics of the World Medical Association (Declaration of Helsinki).

A retrospective analysis included all patients who presented to an academic-based electrodiagnostic laboratory for evaluation from January 2013 to June 2019. Inclusion criteria required three comparative NCS to be completed at the time of evaluation with associated sensory or sensorimotor symptoms in the ulnar nerve distribution (digit 4 and/or 5). The three acceptable NCS were ulnar motor to abductor digiti minimi (ADM), ulnar motor to first dorsal interossei (FDI), and mixed across the elbow. Upon chart review completion, 135 patients met the original inclusion criteria, however, two of those patients had absent motor responses above the elbow. This left 133 patient charts that would satisfy all data points for statistical analysis and began electrodiagnostic testing with suspected UNE. Amplitudes for the mixed NCS have large standard deviations and thus not reliable to separate disease from control, 1 2 therefore, although these data were collected, they were not thoroughly analyzed, other than a range from 1 to 40 microvolts for the median nerve and 1 to 42 microvolts for the ulnar nerve. Distal latencies, amplitudes, velocities were recorded for the previously mentioned NCS (FDI, ADM, and mixed across the elbow) as well as median motor to abductor pollicis brevis, sensory conductions to digits 1,2,5, and sural as applicable. None of the selected patients were diagnosed with median mononeuropathy at the wrist. Signs of acute denervation (fibrillation potentials) were differentiated between C8-T1 ulnar innervated muscles and C8-T1 nonulnar innervated muscles. There were six patients who had undergone ulnar nerve transposition prior to electrodiagnostic testing.

### Electrodiagnostic Protocol

Although these data were gathered retrospectively, the electrodiagnostic laboratory follows the below protocols for obtaining the NCS discussed.

The ulnar motor to ADM NCS was completed using disk electrodes. The active electrode was placed over the belly of the ADM, the reference placed over the fifth metacarpophalangeal joint, and ground placed on the dorsal aspect of the hand. Stimulation sites were marked with a pen, 8 cm proximally from the active electrode (wrist), 5-cm proximal to the midpoint of the olecranon and medial epicondyle (above elbow), and 5-cm distal to the midpoint of the olecranon and medial epicondyle (below elbow). All stimulation points were measured along the course of the ulnar nerve. Distances between stimulation sites were measured to accurately assess nerve conduction velocity (NCV). The elbow was maintained in 90 degrees of flexion with across elbow distance of 10 cm as described in the multiorganizational summary statement. 10

When performing ulnar motor to FDI, disk electrodes were again utilized, with the active electrode placed along the belly of the FDI muscle at approximately the midpoint between the first and second metacarpals, the reference electrode placed at the first metacarpophalangeal joint, and the ground on the dorsal aspect of the hand. The stimulator was then placed at the same stimulation sites as previously described for ADM with NCV again calculated. This technique was consistent with that described previously in the literature. 12

**Fig. 1:**
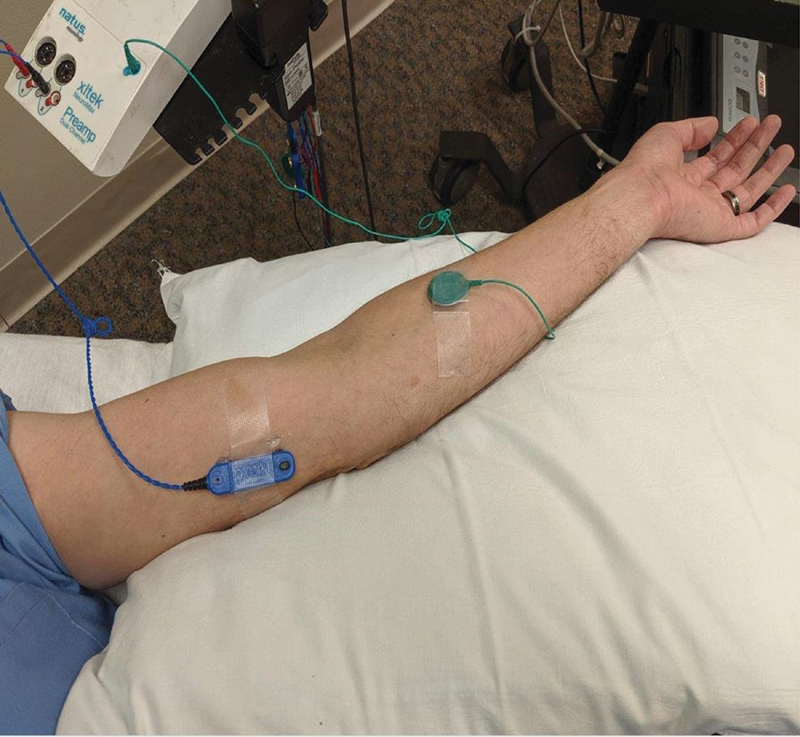
Electrode placement for mixed across the elbow.

To complete the mixed NCS across the elbow, the technique described by Heise and Toledo was used. The bar electrode was applied with the active electrode placed 10-cm proximal to the medial epicondyle between the bellies of biceps and triceps muscles and the ground placed in the mid forearm 2 ( Fig. 1 ). Both median and ulnar nerves were respectively stimulated at the wrist with the elbow extended. The difference between the peak distal latencies for the ulnar and median nerves created the ulnar-median mixed latency difference (U-MLD). This was the number employed to examine for correlation between the NCV across the elbow with the motor NCS to ADM and FDI.

## Results

Of the 135 patient charts, the age range was 18 to 84 years with a mean age of 54 ± 14 years. The sex distribution slightly favored females to males with a ratio of 3:2. Two patients had absent responses to ADM and FDI above the elbow and were subsequently removed for statistical analysis. Of the remaining 133 patients, only 121 of the patients had present responses for the ulnar mixed NCS across the elbow. One patient had absent responses at both ulnar and median mixed NCS across the elbow; this patient was ultimately diagnosed with a length-dependent axonal sensorimotor peripheral polyneuropathy.

**Fig. 2:**
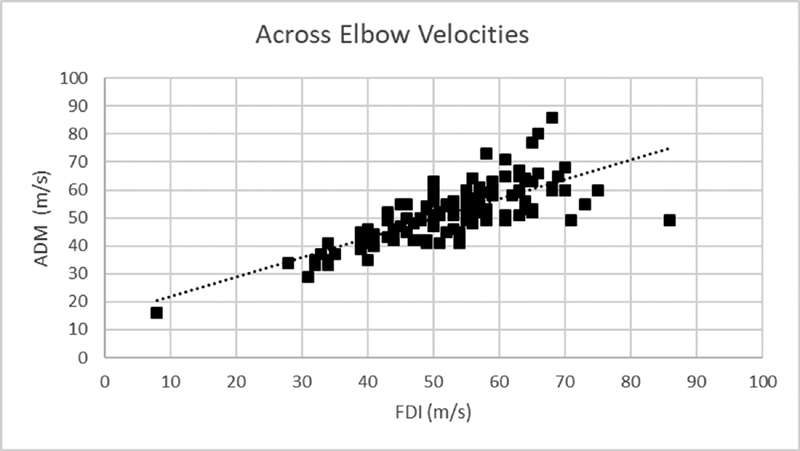
Correlation between ADM and FDI NCV. ADM, abductor digiti minimi; FDI, first dorsal interosseus; NCV, nerve conduction velocities.

**Fig. 3:**
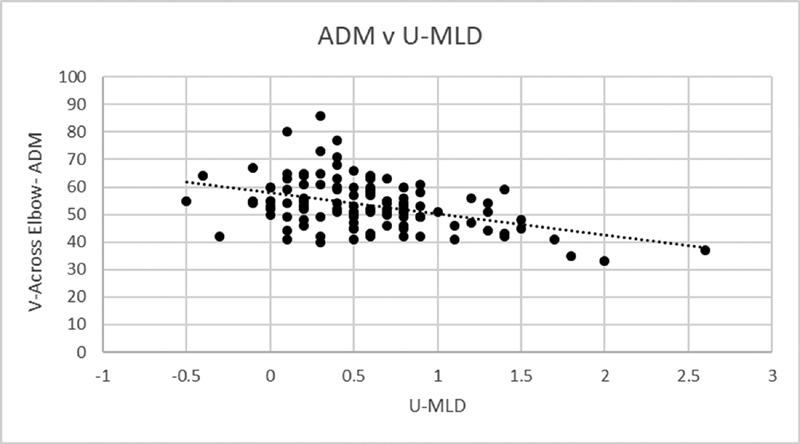
Correlation between ADM NCV and U-MLD. ADM, abductor digiti minimi; NCV, nerve conduction velocities; U-MLD, ulnar-median mixed latency difference.

**Fig. 4:**
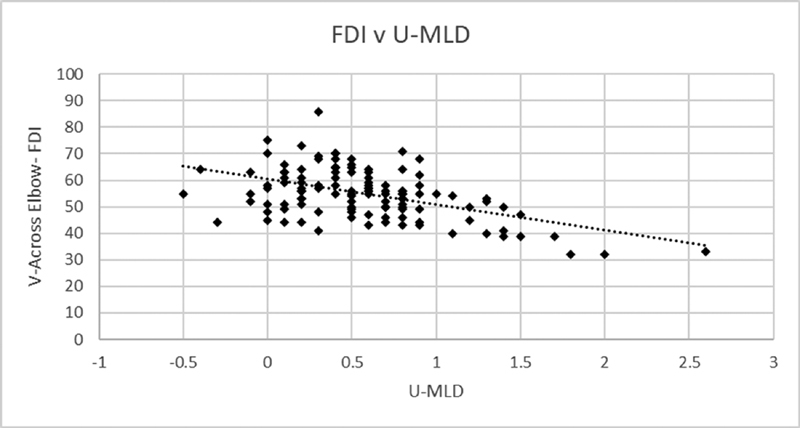
Correlation between FDI NCV and U-MLD. FDI, first dorsal interosseus; NCV, nerve conduction velocities; U-MLD, ulnar-median mixed latency difference.

When assessing for correlation between the two NCV for ADM and FDI, Pearson correlation revealed an *r* = 0.739, *p* < 0.0001 ( Fig. 2 ). There were two different sets of Pearson correlations run for the U-MLD. One comparing U-MLD to across elbow NCV for both ADM and FDI excluding those patients with absent responses for the mixed NCS and the other set that substitutes the largest U-MLD for the absent ulnar mixed responses. Excluding the absent responses, the Pearson correlation between U-MLD to ADM and FDI NCV across the elbow was *r* = −0.479 ( Fig. 3 ) and *r* = −0.543 ( Fig. 4 ), respectively ( *p* < 0.00001).

When the absent responses are replaced with the largest U-MLD, 2.6 ms, the adjusted *r* = −0.60, *p* < 0.00001 compared with ADM and adjusted *r* = −0.642 to FDI, *p* < 0.00001. In both instances, the negative *r* -value indicates the inverse relationship between ulnar velocity across the elbow and U-MLD. That is, the larger U-MLD is associated with a slower ulnar NCV across the elbow.

Attempting to diagnose UNE using NCV across the elbow, leaves the clinician to classify the patient into three groups: clearly normal NCV, clearly abnormal NCV, and borderline NCV. The AANEM (American Association of Neuromuscular and Electrodiagnostic Medicine) criteria of >50 m/s is normal and updated criteria suggests 43 m/s and slower is clearly abnormal. 10 13 Therefore, we considered NCV between 44 and 50 m/s to be borderline. This borderline or indeterminate group comprised 36% of the ulnar nerves assessed in this retrospective study. Of this indeterminate group, 64.5% had an abnormal U-MLD of 0.9 milliseconds or more, only two of which were absent responses (6%). This criterion of 0.9 ms (used by this laboratory) is the midpoint between the less stringent 0.69 ms proposed most recently by Di Virgilio et al 14 and the strict 1.1 ms suggested by Heise and Toledo. 2 We do not attempt to know exactly what number is “abnormal” or truly clinically significant.

## Discussion

This study confirms our hypothesis that there are strong correlations between the three tests for ulnar nerve slowing across the elbow in patients with suspected UNE. The traditional motor studies to ADM and FDI have a stronger correlation. Calculating a *R* ^2^ of approximately 0.54 suggests that these two motor tests agree (or disagree) approximately 50% of the time. When including the absent ulnar mixed nerve responses, the respective *R* ^2^ values between ADM and FDI to U-MLD are 0.36 and 0.41. This suggests that these studies disagree or measure differences approximately 59 to 64% of the time. Although these tests are strongly correlated, they are not identical. This difference provides evidence to use an additional test that measures different aspects of the ulnar nerve. We purposefully did not attempt to calculate sensitivity, specificity, or determine which test was more accurate. There was no reliable way to define a “Gold Standard” or “reference standard” (clinical, electrodiagnostic, or a combination) for the diagnosis of UNE, especially in a retrospective study.

The multiorganizational summary statement regarding ulnar neuropathy hoped to define specific criteria to aid in the proper diagnosis of UNE. 10 These characteristics include across elbow NCV more than 10 m/s slower than forearm NCV, greater than 20% drop or significant change in morphology in compound motor unit action potential amplitude across the elbow, and absolute NCV <50 m/s across the elbow. 10 Updated criteria indicates that <43 m/s is abnormal for across elbow NCV. 13 By definition, that designates NCV between 44 and 49 m/s are abnormal according to the older criteria 10 but normal by the new criteria. 13 This predicament leaves many patients with indeterminate criteria for diagnosing UNE. In such cases where the data remain inconclusive, NCS to FDI, ulnar inching, and/or needle examination of FDI, and ulnar innervated forearm muscles are recommended. 10 Despite these guidelines, diagnosing ulnar neuropathy remained more challenging than other peripheral nerves. 3 4 The difficulty may at least in part be explained in that NCV slowing >10 m/s across the elbow can be as common as 32 to 36% in asymptomatic individuals. 8 Needle electromyography does not always clarify this because forearm flexors have been demonstrated to be spared despite clear evidence of UNE. 1 4 Others have warned of dangers diagnosing nerve disease using only a single test. 15

Balancing sensitivity and specificity remains problematic. A Danish task force created a novel criterion for diagnosing UNE, hoping to improve accuracy. 6 Scores were assigned based on degree of NCV across elbow and forearm, presence or absence of a difference exceeding 20 m/s for across elbow NCV compared with forearm, and presence or absence of conduction block across the elbow. 6 To improve clinical certainty, additional ulnar motor conduction studies were recommended including adductor pollicis, FDI, and sensory near nerve needle technique. Neither the Danish task force nor the AANEM appear to have considered using the mixed across the elbow NCS to enhance diagnostic accuracy. 6 10 Given that patients with mainly sensory symptoms are more difficult to diagnose with electrodiagnostic testing 1 8 ; further tests to evaluate sensory and motor function should be useful.

Robinson et al 15 have described the value of a summary index when diagnosing carpal tunnel syndrome and not relying on a solitary abnormal test. Our study suggests the U-MLD is frequently abnormal in patients with UNE and likely contributes additional information to the electrodiagnostic evaluation of UNE. The potential to identify UNE early in the disease process, prior to more significant motor involvement has the capability to improve surgical outcomes. 1

Few studies assessing the sensory component of nerve function across the elbow have been completed; those that have, illustrate wide variations in amplitudes of controls, which was thought to limit their utility. 1 2 11 Merlevede et al 11 was the first to describe the mixed CNAP across the elbow to further evaluate UNE. However, two major flaws in this research arose; a lack of power with only ten symptomatic patients and continuing to require elbow flexion as part of the study protocol. Elbow flexion continues to contribute to measurement error. 11 Heise and Toledo 2 later adapted this technique to again measure the mixed CNAP but with the elbow in full extension, forearm supinated, and a sample size of 100 symptomatic patients. In this study, four patients with normal ulnar motor NCS had abnormal CNAP latency difference and motor inching studies, suggesting that ulnar motor studies in isolation are missing the diagnosis of UNE in some patients that could be identified using alternative techniques. 2

In our laboratory, we have incorporated the U-MLD into the routine evaluation of UNE. The U-MLD is relatively easy and quick to obtain, especially in thin patients. This test allows for direct comparison to the median nerve, in the same person, at the same time, and limits some intersubject variability (temperature, genetic physiological differences, and arm length). Anecdotally, we have noted that the ulnar response is frequently absent in patients with more severe slowing across the elbow, axon, loss, or significant conduction block. At this point, there is no clear evidence to suggest one test is superior to the others.

This current study has several limitations. The retrospective nature of analysis causes multiple limitations. These include:

- There was likely powerful selection bias of patients that had all three NCS performed. Many patients that were clearly normal or clearly indicative of UNE on motor NCS were not included because the U-MLD was not completed. The U-MLD was most likely recorded when the diagnosis was in doubt and/or it was “convenient.”
- There is no way to reliably calculate sensitivity, specificity, or determine which of the tests had the best accuracy. We intentionally did not attempt to define a “Gold Standard” or “reference standard” for the diagnosis of UNE.
- Past medical history including diabetes or other medical problems, was not collected or considered as a part of exclusion criteria. Without further research, it is unclear how these disease processes would affect the U-MLD.
- Although all data were collected from the same electrodiagnostic laboratory, not all NCS were completed by the same physician. This has both advantages and disadvantages regarding generalizability. To date, there has been no prospective study examining interrater reliability with the mixed across the elbow NCS.

Although these limitations exist, this research solidifies a strong relationship between the mixed across the elbow (U-MLD) and UNE. The negative *r* -value describes the inverse relationship between ulnar NCV across the elbow and U-MLD, that is, the slower the velocity across the elbow the larger the U-MLD. Mixed across the elbow has moderate to strong correlation with both ADM and FDI NCV. This gives construct validity to the U-MLD. All three tests measure ulnar nerve function slightly differently and without further prospective data, it is unclear which single test is best, creating a state of clinical equipoise. The authors propose that some combinations of ulnar motor to ADM, ulnar motor to FDI, and mixed across the elbow tests may be more accurate than relying on motor studies alone.

## References

[1] Campbell W W Ulnar neuropathy at the elbow Muscle Nerve 2000 23 04 450 452 10716754 10.1002/(sici)1097-4598(200004)23:4<450::aid-mus2>3.0.co;2-#

[2] Heise C O Toledo S M Mixed latency difference for diagnosis of ulnar neuropathy at the elbow Arch Phys Med Rehabil 2006 87 03 408 410 16500177 10.1016/j.apmr.2005.11.006

[3] Colorado B S Osei D A Prevalence of carpal tunnel syndrome presenting with symptoms in an ulnar nerve distribution: a prospective study Muscle Nerve 2019 59 01 60 63 30051917 10.1002/mus.26310 PMC8175011

[4] Beekman R Van Der Plas J P Uitdehaag B M Schellens R L Visser L H Clinical, electrodiagnostic, and sonographic studies in ulnar neuropathy at the elbow Muscle Nerve 2004 30 02 202 208 15266636 10.1002/mus.20093

[5] Alrajeh M Preston D C Neuromuscular ultrasound in electrically non-localizable ulnar neuropathy Muscle Nerve 2018 58 05 655 659 29981241 10.1002/mus.26291

[6] Pugdahl K Beniczky S Wanscher B Neurophysiological localisation of ulnar neuropathy at the elbow: validation of diagnostic criteria developed by a taskforce of the Danish society of clinical neurophysiology Clin Neurophysiol 2017 128 11 2205 2210 28972898 10.1016/j.clinph.2017.08.018

[7] Landau M E Diaz M I Barner K C Campbell W W Changes in nerve conduction velocity across the elbow due to experimental error Muscle Nerve 2002 26 06 838 840 12451611 10.1002/mus.10259

[8] Logigian E L Villanueva R Twydell P T Electrodiagnosis of ulnar neuropathy at the elbow (Une): a Bayesian approach Muscle Nerve 2014 49 03 337 344 23716377 10.1002/mus.23913

[9] Robinson L R Micklesen P J Wang L Optimizing the number of tests for carpal tunnel syndrome Muscle Nerve 2000 23 12 1880 1882 11102914 10.1002/1097-4598(200012)23:12<1880::aid-mus14>3.0.co;2-a

[10] American Association of Electrodiagnostic Medicine, American Academy of Neurology, American Academy of Physical Medicine and Rehabilitation.Practice parameter for electrodiagnostic studies in ulnar neuropathy at the elbow: summary statement. American Association of Electrodiagnostic Medicine, American Academy of Neurology, American Academy of Physical Medicine and Rehabilitation Muscle Nerve 1999 22 03 408 411 10086904 10.1002/(sici)1097-4598(199903)22:3<408::aid-mus16>3.0.co;2-7

[11] Merlevede K Theys P van Hees J Diagnosis of ulnar neuropathy: a new approach Muscle Nerve 2000 23 04 478 481 10716756 10.1002/(sici)1097-4598(200004)23:4<478::aid-mus4>3.0.co;2-4

[12] Buschbacher R M Bayindir O Malec J Akyuz G Ulnar motor study to first dorsal interosseous: best reference electrode position and normative data Muscle Nerve 2015 52 02 231 233 25407952 10.1002/mus.24524

[13] Chen S Andary M Buschbacher R Electrodiagnostic reference values for upper and lower limb nerve conduction studies in adult populations Muscle Nerve 2016 54 03 371 377 27238640 10.1002/mus.25203

[14] Di Virgilio G Grapperon A M Fayerstein J Ulnar neuropathy at the elbow: reappraisal of the wrist-upper arm latency difference between ulnar and median nerves Clin Neurophysiol 2020 131 02 372 376 31865138 10.1016/j.clinph.2019.11.022

[15] Robinson L R Micklesen P J Wang L Strategies for analyzing nerve conduction data: superiority of a summary index over single tests Muscle Nerve 1998 21 09 1166 1171 9703442 10.1002/(sici)1097-4598(199809)21:9<1166::aid-mus7>3.0.co;2-5

